# Tendon end separation with loading in an Achilles tendon repair model: comparison of non-absorbable vs. absorbable sutures

**DOI:** 10.1186/s40634-017-0101-9

**Published:** 2017-07-21

**Authors:** Michael R. Carmont, Jan Herman Kuiper, Karin Grävare Silbernagel, Jón Karlsson, Katarina Nilsson-Helander

**Affiliations:** 1 Department of Orthopaedic Surgery, Princess Royal Hospital, Shrewsbury & Telford Hospital, Telford, UK; 2 Department of Biomechanical Engineering, The Robert Jones & Agnes Hunt District General Hospital, University of Keele, Keele, UK; 3 Department of Physiotherapy, University of Delaware, Delaware, USA; 4 Department of Orthopaedic Surgery, Sahlgrenska Academy, University of Gothenburg, Gothenburg, Sweden; 5 Department of Orthopaedic Surgery, Kungsbacka Hospital, Kungsbacka, Sweden

**Keywords:** Tendon rupture, Suture, Biomechanical

## Abstract

**Background:**

Rupture of the Achilles tendon often leads to long-term morbidity, particularly calf weakness associated with tendon elongation. Operative repair of Achilles tendon ruptures leads to reduced tendon elongation. Tendon lengthening is a key problem in the restoration of function following Achilles tendon rupture. A study was performed to determine differences in initial separation, strength and failure characteristics of differing sutures and numbers of core strands in a percutaneous Achilles tendon repair model in response to initial loading.

**Methods:**

Nineteen bovine Achilles tendons were repaired using a percutaneous/minimally invasive technique with a combination of a modified Bunnell suture proximally and a Kessler suture distally, using non-absorbable 4-strand 6-strand repairs and absorbable 8-strand sutures. Specimens were then cyclically loaded using phases of 10 cycles of 100 N, 100 cycles of 100 N, 100 cycles of 190 N consistent with early range of motion training and weight-bearing, before being loaded to failure.

**Results:**

Pre-conditioning of 10 cycles of 100 N resulted in separations of 4 mm for 6-strand, 5.9 mm for 4-strand, but 11.5 mm in 8-strand repairs, this comprised 48.5, 68.6 and 72.7% of the separation that occurred after 100 cycles of 100 N. The tendon separation after the third phase of 100 cycles of 190 N was 17.4 mm for 4-strand repairs, 16.6 mm for 6-strand repairs and 26.6 mm for 8-strand repairs. There were significant differences between the groups (*p* < 0.0001). Four and six strand non-absorbable repairs had significantly less separation than 8-strand absorbable repairs (*p* = 0.017 and *p* = 0.04 respectively).

The mean (SEM) ultimate tensile strengths were 4-strand 464.8 N (27.4), 6-strand 543.5 N (49.6) and 8-strand 422.1 N (80.5). Regression analysis reveals no significant difference between the overall strength of the 3 repair models (*p* = 0.32) (4 vs. 6: *p* = 0.30, 4 vs. 8: *p* = 0.87; 6 vs. 8: *p* = 0.39). The most common mode of failure was pull out of the Kessler suture from the distal stump in 41.7% of specimens.

**Conclusion:**

The use of a non-absorbable suture resulted in less end-to-end separation when compared to absorbable sutures when an Achilles tendon repair model was subject to cyclical loading. Ultimate failure occurred more commonly at the distal Kessler suture end although this occurred with separations in excess of clinical failure. The effect of early movement and loading on the Achilles tendon is not fully understood and requires more research.

## Background

Rupture of the Achilles tendon leads to long-term morbidity, particularly 10–30% calf weakness (Barfod et al., 2017; Horstmann et al., 2012; Lantto et al., 2015; Mavrodontidis et al., 2015). This weakness has been associated with tendon elongation (Silbernagel et al., 2012). Operative repair of Achilles tendon ruptures leads to improved early outcome (Keating & Will, 2011), in terms of strength (Lantto et al., 2016; Willits et al., 2010) and functional activities (Olsson et al., 2011; Olsson et al., 2014) and reduced tendon elongation of 18.7 mm compared to non-operative treatment (*P* < 0.01) (Heikkinen et al., 2017) although lengthening (5 mm at 12 months, and 14.5 mm at 13 years) still occurs even in augmented repairs (Heikkinen et al., 2016; Pajala et al., 2009).

**Fig. 1:**
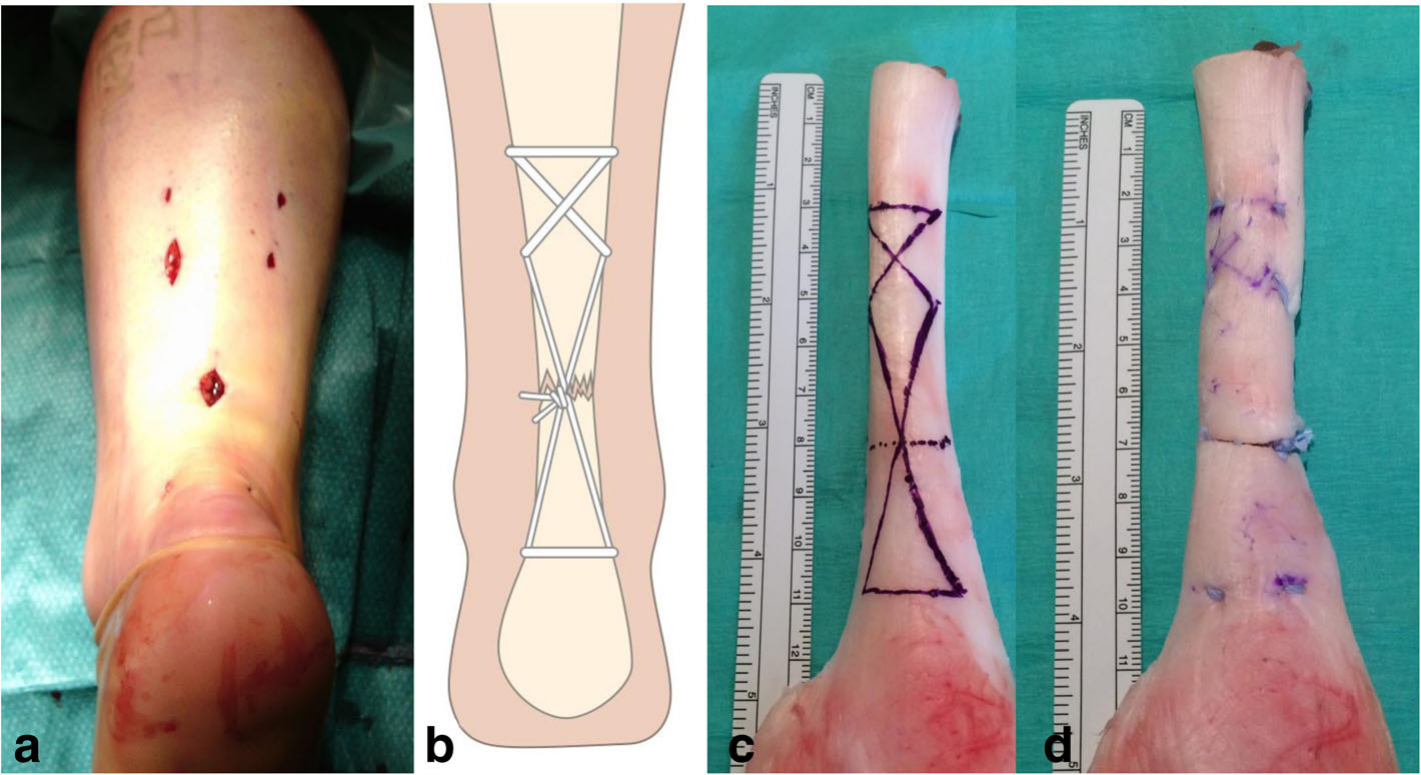
**a** The cosmetic and wound size of minimally invasive repair prior to skin closure, **b** a schematic of repair performed using the combination of a modified Bunnell suture proximally and a Kessler suture distally. **c** Bovine flexor tendon following dissection of the fascia and paratenon layers. **d** The final testing specimen of an Achilles tendon repair model. Note the end-to-end tendon apposition

Minimally invasive and percutaneous surgery for the Achilles tendon rupture aims to provide a stable repair, allowing early weight-bearing and movement, whilst minimizing the risks of wound breakdown or infection (Del Buono et al., 2014; Khan & Carey Smith, 2010; McMahon et al., 2011; Yang et al., 2017) (Figs. 1a. Biomechanical comparison has shown that the Ma and Griffiths percutaneous technique (Ma & Grifith, 1977) has approximately 50% of the strength of open repairs and has an increased iatrogenic nerve injury rate (Chan et al., 2011; Del Buono et al., 2014; Hockenbury & Johns, 1990) although this has been reduced using of absorbable sutures and visualization and protection of the nerve during surgery (Klein et al., 1991).

The use of a combined modified Bunnell and Kessler suture configuration (Carmont & Maffulli, 2008) (Fig. 1b) using an absorbable monofilament suture shows good clinical outcome (Al-Mouazzen et al., 2005; Carmont et al., 2013) as well as specific patient subsets; athletes (Maffulli et al., 2011b), the elderly (Maffulli et al., 2010) and diabetic patients (Maffulli et al., 2011c). This suture technique has similar repair strength to that of the box suture formed using the Achillon device (Longo et al., 2012).

Although monofilament absorbable sutures e.g. polyglyconate co-polymer Maxon (Covidien, Mansfield, MA, USA) are broken down over 6 weeks, which may potentially avoid stress shielding (Table 1), the associated inflammatory response may actually weaken the healing tendon predisposing to re-rupture at a key phase in the rehabilitation process. Braided non-absorbable sutures made from polyester coated with a polytetrafluoroethylene (PTFE) layer (Fiberwire®, Arthrex, Naples, USA) are commonly used for Achilles tendon repair (De La Fuente et al., 2016a; Hsu et al., 2015; Keller et al., 2014). The sutures offer the advantages of strength, smooth passage through the tendon and secure knots (Benthien et al., 2006). An increased number of core suture strands has been shown to increase the strength of suture repair models (Backus et al., 2017; Ortiz et al., 2012) but this has not been studied using the combined modified Bunnell and Kessler configuration.

**Table 1:** Characteristics of Fiberwire and Maxon sutures

Suture Configuration	Fiberwire®	Maxon®
Size USP	2	1
Single strand diameter/mm	0.5	0.4
Mass suture Cross Sectional Area/mm^2^	4-strand = 0.54 6-strand = 0.8	8-strand = 0.69
Material	Polytetrafluoroethylene (PTFE) coated polyester	Polyglyconate co-polymer of glycolic acid and Trimethylene Carbonate
Thread type	Braided	Mono-filament
Colour	Blue	Green
Absorption	Non-absorbable	Absorbable predictable 180 days
Ultimate tensile strength single strand	345 N	54 N Strength profile: 80% at 1 week 75% at 2 weeks 65% at 3 weeks 50% at 4 weeks 25% at 6 weeks

The aim of this biomechanical study was to determine differences in initial separation, strength and failure characteristics of differing sutures and numbers of core strands in a percutaneous Achilles tendon repair model. The null hypothesis was that there would be no difference in tendon separation between repairs when subject to cyclical loading.

## Methods

Prior to commencing the study, approval was obtained from the Robert Jones and Agnes Hunt Hospital Research and Development Department (RL1 620 IRAS 173197). All specimens were obtained from local abattoirs from juvenile animals (2 years old) slaughtered for human consumption. Pilot testing was performed using 5 ovine tendons and 5 bovine tendons. This determined that ovine tendons were too small to insert the suture materials in the in vivo configuration and as a result bovine tendons were used. Subsequent testing of bovine tendons permitted consistent repairs to be made to allow cyclical loading to ultimate tensile failure and minimize clamp pull out. Bovine tendons have previously been successfully used to provide specimens to represent human Achilles tendons for biomechanical studies (Benthien et al., 2006; De La Fuente et al., 2016b; Guzzini et al., 2017; Ortiz et al., 2012).

### Specimen preparation

Nineteen fresh frozen lower limb flexor tendons were prepared following thawing in a refrigerator. The flexor tendon specimens were resected proximal to the musculotendinous junction and the collateral insertional slips from either side of the calcaneus. The layers of the fascia and the paratenon were removed and the distal muscle bulk was resected off the septum. The mid-substance flexor tendon specimen sample was typically 10-12 cm in length. The thinnest section of the tendon was marked with a transverse line using a surgical marking pen for the tenotomy mean (SD) thickness antero-posterior (AP) was 13.6 mm (1.6) (range 10.8–17.0 mm), transverse thickness using a vernier caliper and the cross sectional area determined as 196.5mm^2^ (30.4) (range 131.97–245.9) using the formula:

$$ \mathrm{Area}=\uppi \bullet \mathrm{AP}\ \mathrm{radius}\bullet \mathrm{Transverse}\ \mathrm{radius}. $$

Additional transverse marks, at 3 cm and 5 cm proximally and another mark at 3 cm distally to the tenotomy line indicated the transverse and oblique lines of suture passage (Fig. 1c). A number 10 scalpel was then used to make a transverse tenotomy representing the rupture site. The insertion point into both side of the calcaneus in the bovine specimens made it difficult to measure corresponding a typical rupture site 4-6 cm proximal to the insertion as in humans so the tenotomy was made at the thinnest part of the tendon.

A 9 cm B204/00 curved needle with a cutting tip was used for all suture passes (Acufirm, Ernst Kratz GmbH), threaded with 2, 3 or 4 sutures. A modified Bunnell suture was inserted through the proximal tendon 5 cm from the tenotomy, approximately a 3 mm distance was maintained between the site of the emerging needle and the position of re-insertion. Distally, a Kessler suture was formed by transversely passing the passing the needle at 3 cm distal to the tenotomy (Fig. 1c & d). Sutures from either end of the tendon were then tied, en mass, using a double throw surgeons knot and 4 subsequent hitches as tight as possible with the aim of apposing the part of the tenotomised tendon ends despite the interposed suture knots (Fig. 1d).

The tendon ends were then compressed in a vice and wrapped in course sandpaper mesh before being loaded into the clamp and tightened. An initial tension of 10 N was applied using a testing machine and the antero-posterior diameter and transverse diameter of the tendon measured using a vernier caliper, in millimeters to two decimal places (Digitromic Caliper, Design Line Series, Moore & Wright, UK). These measurements were repeated several times until a consistent value was obtained. If apposition of the tendon ends occurred, separation was determined as zero, otherwise the initial separation was measured using the caliper. This same position on the tendon was used for all subsequent separation measurements.

Specimens were tested in random order to allow for optimization of surgical technique. In Group 1, 6 specimens were prepared using 4-strand Fiberwire sutures. In Group 2, 7 specimens were repaired using 6-strand Fiberwire sutures. Finally 6 specimens were repaired using 8-strand Maxon sutures. These suture numbers and combinations were chosen as they have been used in standard practice (Carmont et al., 2013; Carmont et al., 2015; Carmont et al., 2017). There was no significant difference in cross sectional area between the groups (*p* = 0.247).

### Loading protocol

**Fig. 2:**
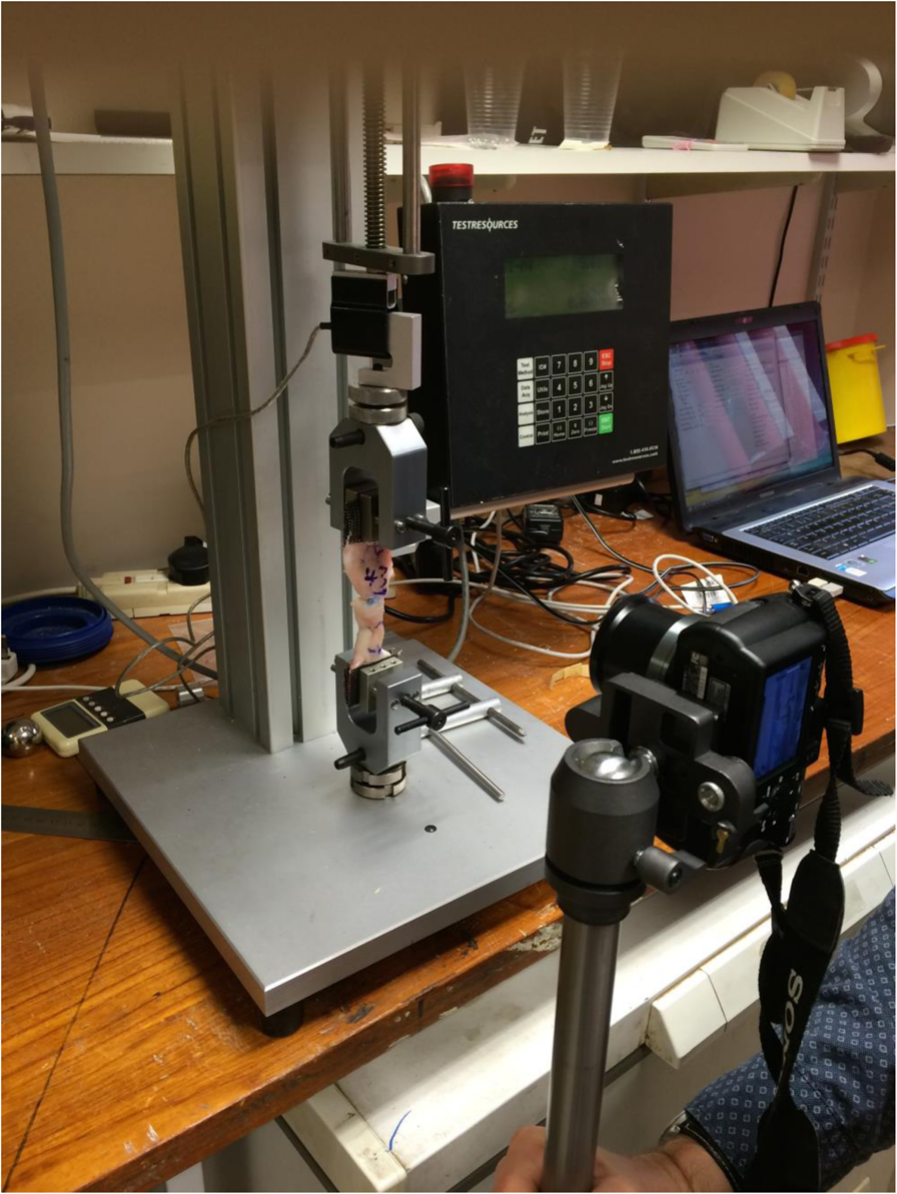
The biomechanical testing jig and experimental set up

Tendon specimens were subject to a cyclical loading programme, using a hydraulic biomechanical testing machine (ESH, Brierley Hill, UK) and the output sampled using an analogue to digital converter connected to a laboratory computer (Fig. 2). This programme has been chosen as it has been used in testing Achilles tendon repair models to represent the early stages of rehabilitation (Akizuki et al., 2001; Demetracopoulos et al., 2014; Lee et al., 2009): passive ankle range of motion (100 N) and walking in a controlled ankle motion walking boot with a 2.5 cm heel lift (190 N). The present study included 4 phases of cyclical loading:

1. “Pre-conditioning” with 10 cycles at 100 N at frequency 1 Hz.
2. An additional sequence of 90 cycles at 100 N.
3. A further 100 cycles at 190 N.
4. Finally the tendon was loaded until complete failure.

After each phase of loading the separation of the tendon ends was measured using the vernier caliper (Ortiz et al., 2012). Clinical failure was considered to be an end-to-end separation of greater or equal to 20 mm, comparable to elongation noted in non-operative management (Heikkinen et al., 2017). If a phase of loading could not be completed the number of cycles that were completed before failure was noted.

Additionally the mode of failure of each repair model was noted. Cyclical loading of the tendon was digitally recorded using a camera to determine the specific characteristics and pattern of the loading and failure of the tendon repair model.

### Statistical analysis

A sample size of six (*n* = 6) is the standard sample size recommended for fatigue testing (ASTM E739–91, 1998). This was consistent with group sample numbers for other Achilles tendon biomechanical studies: Demetracopoulos *n* = 7, Lee *n* = 5, Longo *n* = 9, Ortiz *n* = 5) (Demetracopoulos et al., 2014; Lee et al., 2009; Longo et al., 2012; Ortiz et al., 2012).

Separation measurements between groups over the 4 phases of the loading protocol were compared using one-way ANOVA. Censored regression analysis was performed to evaluate ultimate failure to account for specimen pull out from the jig. Data was analyzed using SPSS Version 24. A level of significance of *p* < 0.05 was chosen.

## Results

In 79% of specimens the suture configuration permitted end-to-end tendon apposition when positioned on the testing jig and subjected to an initial load of 10 N (Table 2). Two specimens in each of the 4-strand and 8-strand groups had initial separation rather than apposition due to the interposed knots between the tendon ends. Only one specimen in the 6-strand group did not have initial end-to-end apposition, however, this was the largest at 3.9 mm separation.

**Table 2:** The separations of the Achilles tendon repair models with the 4 phases of loading, and the mode of failure

Specimen number	Strand	XSectional Area/mm ^2^	Initial Separation (10 N)/mm	Separation (10 cycles 100 N)/mm	Separation (100 cycles 100 N)/mm	Separation (100 cycles 190 N)/mm	Ultimate Tensile Failure/N	Mode of Failure
1	4	143.3	2.9	6.8	10.3	20.5	241	Pull out jig
2	4	170.5	0	6.7	9.3	17.0	405	Distal pull out
3	4	192.8	0	3.1	5.3	15.4	399	Knot Failure
4	4	222.3	0	7.1	10.2	19.6	411	Pull out jig
5	4	166.3	3.0	6.0	8.6	16.6	464	Suture snap
6	4	188.3	0	5.6	7.4	15.3	508	Distal pull out
Mean(SD)			1.0(1.5)	5.9(1.5)	8.5(1.9)	17.4(2.2)	465(27)	
1	6	221.4	3.9	7.7	13.7	23.5	321	Pull out jig
2	6	131.9	0	2.6	5.4	12.8	419	Proximal pull out
3	6	245.9	0	2.5	3.4	9.4	462	Pull out jig
4	6	173.8	0	5.4	8.7	17.7	567	Distal pull out
5	6	193.8	0	3.7	5.1	13.5	464	Knot Failure
6	6	214.5	0	2.6	14.5	16.3	638	Pull out jig
7	6	207.2	0	3.6	6.9	17.3	506	Pull out jig
Mean(SD)			0.6(1.5)	4.0(1.9)	8.2(4.3)	15.8(4.5)	543(50)	
1	8	207.8	1.8	10.8	13.8	27.1	286	Pull out jig
2	8	212.6	0	8.5	10.7	21.6	506	Distal pull out
3	8	176.3	0	12.6	20.2	Failed		Knot failure
4	8	229.8	0	12.4	17.7	Failed		Knot failure
5	8	201.1	2.4	14.8	19.1	31.7	591	Distal pull out
6	8	233.6	0	10.0	13.3	26.2	526	Pull out jig
Mean(SD)			1.1(1.4)	11.0(2.7)	14.2(3.5)	26.6(4.1)	422(81)	

The Cross Sectional Area of the Ellipsoid tendons were determined using the formula: Area = π•AP radius•Transverse radius

**Fig. 3:**
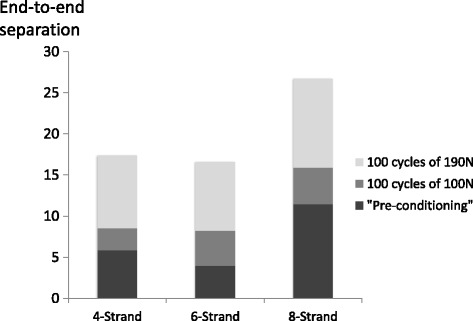
Tendon separations during initial loading (10 N), and after 10 cycles of 100 N (preconditioning), 100 cycles of 100 N and 100 cycles of 190 N

Pre-conditioning of 10 cycles of 100 N resulted in separations of 4 mm for 6-strand, 5.9 mm for 4-strand but 11.5 mm in 8-strand repairs, this comprised 48.5, 68.6 and 72.7% of the separation that occurred after 100 cycles of 100 N (Fig. 3).

The tendon separation after the third phase of 100 cycles of 190 N was 17.4 mm for 4-strand repairs, 16.6 mm for 6-strand repairs and 26.6 mm for 8-strand repairs. Over the four phases of the loading programme there was a significant difference between the different repairs (*p* < 0.0001). Four and six strand non-absorbable repairs had significantly less separation than 8-strand absorbable repairs (*p* = 0.017 and *p* = 0.04 respectively). There were no significant differences between the 4 and 6 strand repairs (*p* = 1). When clinical failure was considered to be tendon end separation of ≥20 mm all of the 8-strand repairs failed, whereas only one of the 4-strand specimens and none of the 6-strand specimens failed.

Out of the 19 specimens tested, 7 of these resulted in pull out from the clamps holding the tendons before cyclical loading resulted in ultimate failure of the repair. Regression analysis reveals no significant (*p* = 0.32) difference between the ultimate strength of the 3 repair models (4 vs. 6: *p* = 0.30, 4 vs. 8: *p* = 0.87; 6 vs. 8: *p* = 0.39). The mean ultimate tensile strengths (SEM) were for 4-strand 464.8 N (27.4), 6-strand 543.5 N (49.6), and 8-strand 422.1 N (80.5). In all specimens ultimate tensile failure occurred at separation far in excess of clinical failure.

The mode of failure was distal pull out in 41.7% of specimens, proximal pull out in 20.8% of specimens and knot failure in 33.3% of specimens. Knot failure occurred in two absorbable monofilament suture repairs and one of the 4 and 6 strand specimens. There was only one case (in a 4-strand repair), in which the suture snapped.

## Discussion

The most important finding of this study was that repairs performed with non-absorbable braided sutures resulted in less separation than with absorbable monofilament sutures when subjected to a loading protocol.

The separation of tendon ends when subjected to 100 cycles of 100 N loads was less than 1 cm when specimens were repaired with the non-absorbable suture. When loads of 190 N were applied for 100 cycles, the non-absorbable repair models had 16.5 mm of separation whereas the absorbable suture repairs resulted in clinical failure (≥20 mm) or actual failure.

Although the two loading protocols are considered to be consistent with walking in a brace with a 2.5 cm heel raise and passive range of movement exercises, the quantitative separation reported in this study is not directly applicable to patients for number of reasons. Firstly the bovine tendons were larger than those found in patients, they did not have pre-existing degeneration (Józsa & Kannus, 1997; Maffulli et al., 2011a) and technically an open repair rather than a percutaneous repair of the dissected specimens was performed. This study was to compare suture configuration inserted using a percutaneous/minimally invasive technique. The differing superficial soft tissues between human and bovine tendons necessitated that specimens were dissected free from superficial soft tissues. The sutures were subsequently placed as an open repair eliminating the confounding variable of poor suture placement, which can occur in using a percutaneous placement (Demetracopoulos et al., 2014; Heitman et al., 2011). In Heitmann’s series, 3/10 specimens had sutures, which passed superficial to the tendon when the Achillon jig was used to guide suture passage (Heitman et al., 2011). The favourable separation and failure characteristics of the 6-strand non-absorbable suture over the 8-strand absorbable suture shown in this study can, however, be applied to the management of Achilles tendon ruptures in patients.

**Fig. 4:**
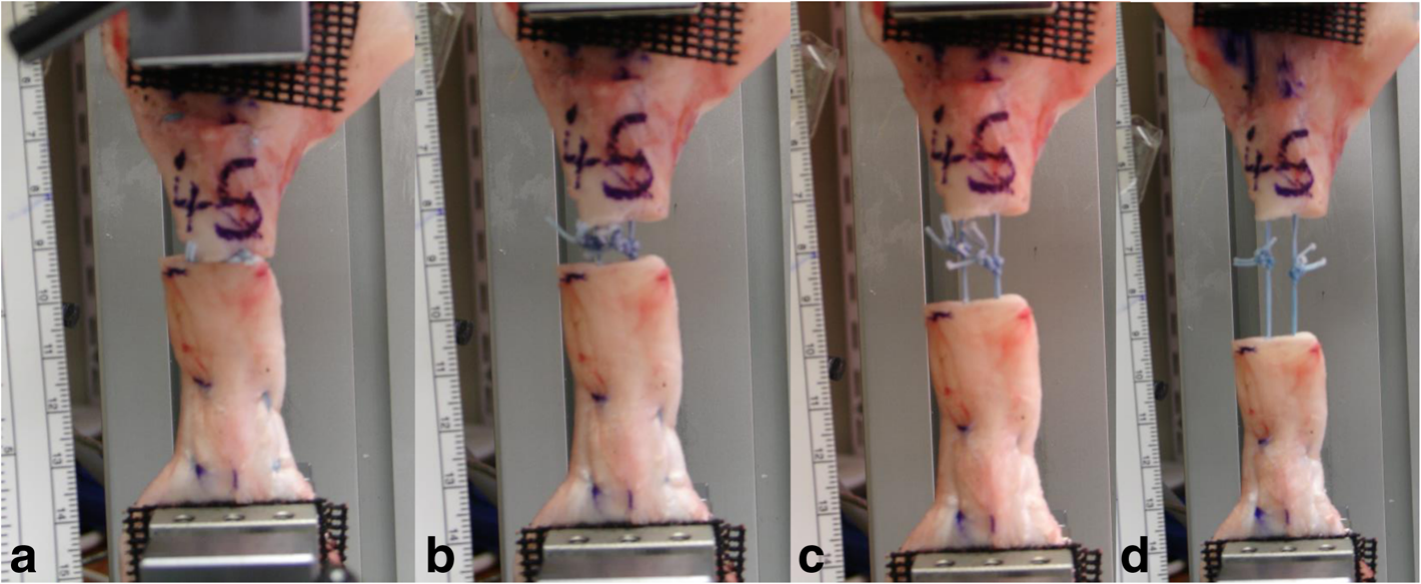
A tendon specimen repaired with a non-absorbable suture during **a** initial loading (10 N) note the end to end apposition, and after **b** pre-conditioning (10 cycles of 100 N), **c** 100 cycles of 100 N and **d** 100 cycles of 190 N

**Fig. 5:**
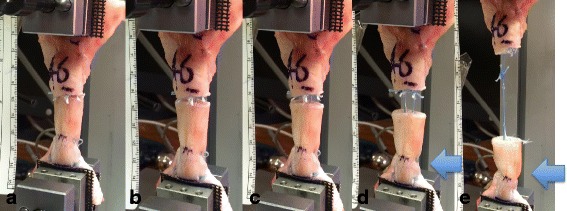
The tendon specimen repaired with a non-absorbable suture during **a** initial loading (10 N), and after **b** pre-conditioning (10 cycles of 100 N), **c** 100 cycles of 100 N and **d** 100 cycles of 190 N demonstrating knot failure. **e** Ultimate tensile failure﻿. *Arrows* indicate the reduction in length and increase in thickness related to the Bunnell suture termed the accordion effect

**Fig. 6:**
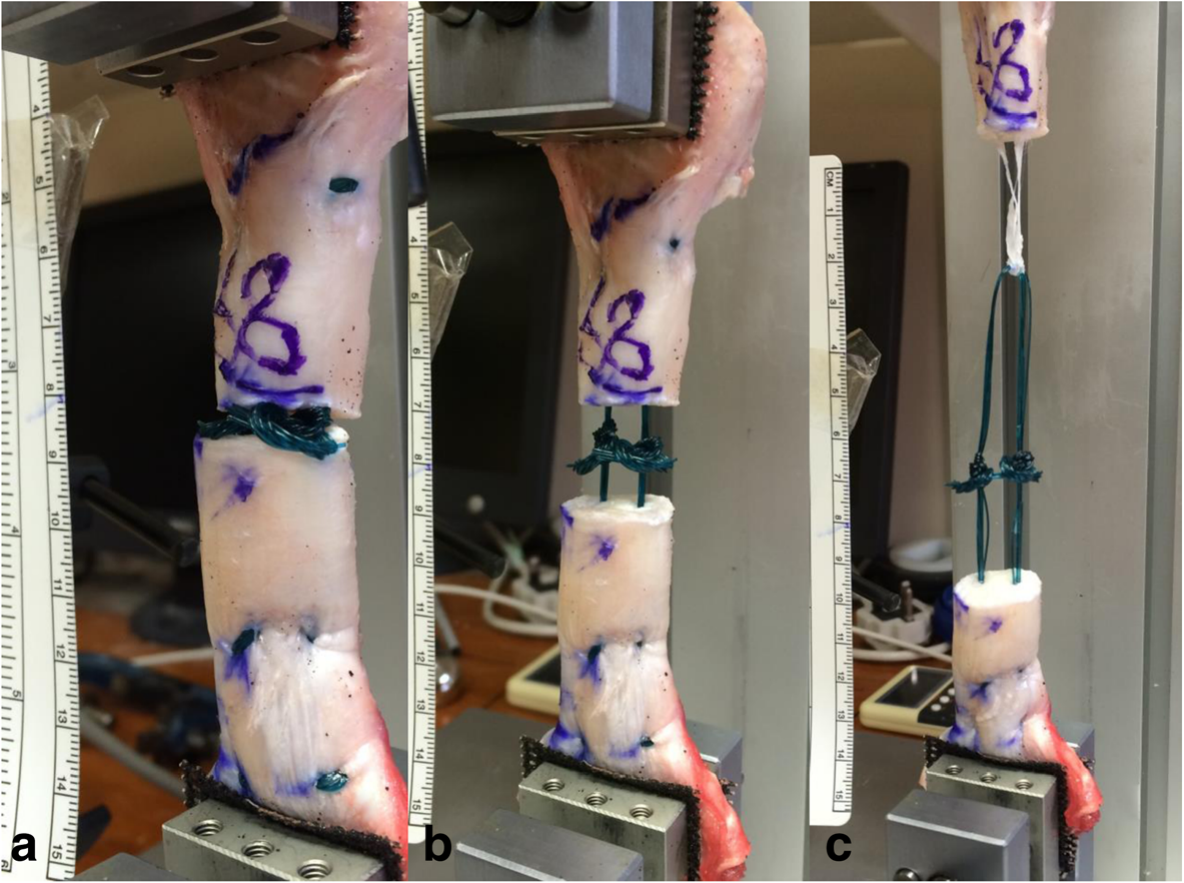
A tendon specimen repaired with absorbable suture with **a**) initial loading, **b**) 100 cycles of 190 N. Ultimate failure occurring by distal pull out with separation in excess of clinical failure (**c**)

Another finding of the study was the importance of “pre-conditioning” of the suture configuration prior to knot tying. In this study approximately half of the separation (68.6% 4-strand, 48.5% 6-strand and 72.7% 8-strand) occurred during the first 10 cycles of 100 N (Figs. 4b, 5b & 6b), compared to the separation that had occurred by 100 cycles (Figs. 4c, 5c & 6c). The aspect of pre-conditioning was highlighted in Clanton’s study when 9.5 mm 77.9% of the overall 12.2 mm elongation occurred during the first 10 of 250 cyclic loading of 20-100 N (Clanton et al., 2015). By comparison when pre-conditioning was applied prior to knot tying Demetracopoulos found a 1000 cycles 20-100 N were required to produce 5 mm of separation in Achillon and PARS repairs (Demetracopoulos et al., 2014). It is recommended that pre-conditioning of at least 10 firms pulls are applied to the sutures in the proximal and distal tendon ends before the sutures are knotted during operative repair.

The use of the digital camera permitted the characteristics of failure to be observed and commented upon. With increased loading, usually in the loading to failure phase, the proximal tendon shortened slightly and appeared to bulk up as the suture tightened, the accordion effect (Figs. 4, 5 & 6). In vivo, the consequences of this shortening effect may be minimized by pre-conditioning and then ensuring tendon end-to-end apposition with knots tied with the ankle placed in plantar flexion compared to the non-injured ankle. This operative “over-tightening” compensates by subsequent elongation with loading, movement and remodeling (Heikkinen et al., 2016; Pajala et al., 2009; Schepull et al., 2012; Silbernagel et al., 2012). Another biomechanical property observed was that the absorbable sutures were notably more elastic than the non-absorbable sutures with more stretching and recoil rather than that observed with the stiffer non-absorbable suture. This elastic nature of absorbable monofilament repair may be functionally beneficial in avoiding stress shielding of the repair although this must be considered against knot stability and pre-conditioning characteristics.

In terms of ultimate failure of the repair models, the 6-strand non-absorbable repair had the highest ultimate tensile failure. It is interesting to note that clinical failure, ≥ 20 mm of separation occurred in all specimens prior to ultimate tensile failure. Twenty millimeters of separation was chosen as this was the tendon lengthening noted to occur following non-operative treatment in Heikkinen’s study (Heikkinen et al., 2017). Such lengthening would confer no advantage over operative treatment. The commonest mode of failure was distal pull-out of the Kessler suture in 41.7% of cases, in concert with other reported failure patterns (Longo et al., 2012). Thirty three percent of failure was due to knot failure and half of these (*n* = 2) occurred in the absorbable 8-strand suture. The tying of the 8-strand monofilament knots noticeably had greater friction than the PTFE coated braided sutures. One hitch of absorbable suture was noted to untie during the loading sequence although knot failure occurred equally in absorbable and non-absorbable sutures. In vivo, the suture knots are surrounded by healing tendon end and so may be less prone to unraveling with movement at the 2-week time point.

There are a few limitations to this study although many of these relate to the tenotomy model common to many biomechanical aspects of Achilles tendon research.

The first is the problem of holding the specimens firmly in a clamp during the cyclical loading programme. Despite pilot testing to reduce tendon-clamp interface failure, loading of 7 out of the 19 specimens lead to pull out from the clamp. Four of these occurred at loads ≥400 N, however, all had failed clinically (≥20 mm separation) by this point.

The use of a “clean” tenotomy, with a flat tendon end, may prevent shortening of the tendon at the time of repair due to the knots “within” the repair site. Artificial mop ends have been applied to tenotomy models (Backus et al., 2017; Grieco et al., 2015), which may permit shortening of the tendon by mop end-to-mop end apposition during repair and so subsequent separation with loading would restore normal length. In this model however, the flattened tendon ends permitted more accurate measurement of gapping measurement. The absence of a stable calcaneus may have also prevented pre-conditioning and the repair from being in as much tension as possible.

Suture placement in dissected tendons allowed optimal suture placement and clear looping of the suture outside the tendon. In vivo, the needle may pass down the same suture passage weakening the repair or even permit interposed fascia and paratenon layers forming adhesions reducing clinical outcome. Although monofilament is considered to slide through the tendon tissue more smoothly, it was considered to have greater friction between the strands than the PTFE coated braided suture. This may have reduced tension throughout the suture configuration and knot tightness.

This time zero model does not account for the healing of the tendon with time. It does, however, suggest that the repair may permit the maintenance of tendon tension during early loading whilst healing. The suture configurations tested were able to prevent gapping and excess lengthening; suggesting they are stable enough to allow early protected weight bearing on the metatarsal heads using a protective dorsal plantar flexion shell and axillary crutches and active movement exercises (Carmont & Maffulli, 2008; Carmont et al., 2013; Carmont et al., 2015; Carmont et al., 2017). De La Fuente has recently demonstrated that functional braces should hold the ankle in 14° plantar flexion to prevent failure (De La Fuente et al., 2016c). Clinically tendons repaired using these sutures and configurations did have an increase in Achilles Tendon Resting Angle (ATRA) following rehabilitation despite intra-operative shortening (Carmont et al., 2015; Carmont et al., 2017). At time zero an end-to-end separation of greater than 4 mm (Ecker et al., 2016) and 1 cm (Hutchison et al., 2015) with passive flexion have been considered as indications for operative repair.

Further research is needed regarding the stability of early repair, avoidance of repair separation, tendon lengthening and yet the maintenance of elasticity to optimize resumption of biomechanical characteristics. The avoidance of tendon lengthening, the reduction in Achilles Tendon Resting Angle and the restoration of normal gastrocsoleus strength and Achilles tendon viscoelastic properties are important aspects is recovery from an Achilles tendon rupture. The pre-conditioning of sutures within the tendon ends, prior to knot tying is recommended to optimise repair tension and minimize tendon end-to-end separation.

## Conclusions

The use of a non-absorbable suture resulted in less end-to-end separation when compared to absorbable sutures when an Achilles tendon repair model was subject to cyclical loading. Ultimate failure occurred more commonly at the distal Kessler suture end although this occurred with separations in excess of clinical failure. The effect of early movement and loading on the Achilles tendon is not fully understood and requires more research.
